# Oropouche Virus (OROV) Vaccine Development for Vulnerable Populations: Epidemiological Context, Challenges and Future Directions

**DOI:** 10.3390/vaccines14030267

**Published:** 2026-03-16

**Authors:** Wenrui Wu, Yiu-Wing Kam

**Affiliations:** Division of Natural and Applied Sciences, Duke Kunshan University, No. 8 Duke Avenue, Kunshan 215316, China; wenrui.wu@dukekunshan.edu.cn

**Keywords:** Oropouche virus, vaccine development, vulnerable populations, arbovirus, emerging infectious diseases, public health surveillance

## Abstract

Oropouche virus (OROV) is an emerging arthropod-borne virus in the Americas that has evolved from a pathogen historically restricted to forest environments into an increasingly important regional and international public health concern. Despite decades of documented circulation, the true burden of OROV infection remains substantially underestimated, largely because of frequent misdiagnosis and the high proportion of asymptomatic or subclinical infections. This review synthesizes current evidence on the historical emergence, epidemiology, transmission dynamics, and clinical features of OROV, with a particular focus on populations at increased risk due to biological susceptibility, environmental exposure, and limited access to healthcare. Drawing on seroepidemiological data, we demonstrate that OROV transmission is far more widespread than routine surveillance suggests and examine how factors such as age, pregnancy, immune status, underlying health conditions, occupational exposure, and healthcare accessibility interact to influence disease risk and detection. Although multiple vaccine platforms have shown promise in preclinical studies, progress toward clinical development remains constrained by limited immunological evidence, shortcomings of available animal models, diagnostic uncertainty, and structural barriers in endemic regions. We propose that future OROV vaccine development prioritize population-specific needs rather than focusing solely on technological platforms, and that effective prevention will require integrating vaccination with strengthened surveillance, improved diagnostics, and equitable delivery systems.

## 1. Introduction

Oropouche virus (OROV) is an arthropod-borne virus (arbovirus) belonging to the order *Bunyavirales*, family *Peribunyaviridae*, and genus *Orthobunyavirus*, specifically classified as the *Orthobunyavirus oropoucheense* species [1]. It possesses a negative-sense, single-stranded RNA genome enclosed in a spherical lipid envelope measuring approximately 80–120 nm in diameter [2,3]. The genome is tripartite, comprising large (L), medium (M), and small (S) segments that encode the RNA-dependent RNA polymerase (RdRp), the surface glycoproteins (Gn and Gc), and the nucleocapsid protein (N), respectively [1]. The advent of molecular methods and next-generation sequencing has led to the determination of full genomic sequences [4]. Genetic studies show that OROV undergoes frequent re-assortment of its tri-segmented RNA genome, a feature that likely facilitates its adaptation to diverse hosts and vectors [5,6,7]. OROV belongs to the Simbu serogroup, which comprises approximately 25 antigenically related viruses grouped into seven complexes and two phylogenetic subclades—Manzanilla and Oropouche (subclade A) and Simbu, Akabane, Sathuperi, Shamonda, and Shuni (subclade B) [8,9].

Historically, OROV was first isolated in 1955 from a febrile patient in Trinidad and Tobago during an outbreak of acute febrile illness, and was later identified in a three-toed sloth (Bradypus tridactylus), suggesting a zoonotic origin [3,10]. The earliest recognized human outbreak occurred in 1961 in the Amazon region of Brazil [11]. These early observations mark the initial recognition of OROV and provide a foundation for understanding its subsequent emergence and spread. Since then, epidemics have been reported in several South and Central American countries, particularly Brazil, Peru, Panama, and Ecuador, causing over 500,000 clinically confirmed cases and likely many more unreported cases due to diagnostic overlap with co-circulating arboviruses such as dengue, Zika, yellow fever, and chikungunya [10,12]. Although the virus was initially confined to tropical rainforest environments, rapid urbanization and climate variability have increasingly favored its expansion into peri-urban settings. Based on available historical and epidemiological evidence, OROV emergence can be broadly conceptualized as comprising four successive phases: discovery, endemic establishment, regional expansion, and recent re-emergence (Table 1).

This review will first detail the historical emergence and epidemiology of OROV, followed by its clinical and public health impact. We then define the populations most vulnerable to infection and analyze the current landscape and specific challenges in developing a protective vaccine. Finally, we propose a strategic framework for vaccine implementation and equitable access, concluding with recommendations for a population-centered development pathway.

This study was conducted as a narrative review. Relevant literature published between 1961 and 2026 was identified through searches of the PubMed and Scopus databases. Articles were selected based on their relevance to Oropouche virus epidemiology, transmission dynamics, vulnerable populations, and vaccine development. Epidemiological data were obtained from reports by the World Health Organization (WHO), its Regional Office for the Americas (the Pan American Health Organization, PAHO), and Brazilian national surveillance reports.

## 2. Epidemiology and Transmission

### 2.1. Epidemiology and Disease Burden

OROV is recognized as one of the most common arboviruses causing acute febrile illness in the Amazon Basin and neighboring regions of South and Central America. Endemic transmission occurs predominantly in northern Brazil, where the virus is responsible for cyclic outbreaks every three to five years [2]. Epidemics typically peak during the rainy season when vector density increases and human exposure is maximized, especially for arboviruses [28]. Because Oropouche fever resembles dengue and Zika clinically, large numbers of cases go undiagnosed or are misclassified [6]. Serological surveys in the Peruvian Amazon showed antibody prevalences of 35% in urban populations, 24–46% in forest communities, and 18% in rural settlements, suggesting continuous OROV transmission across different ecological settings [12]. Model-based risk mapping indicates that deforestation, urban expansion, and climate change may allow northward spread of competent vectors into the Caribbean and possibly southern North America [29]. In 2025, the World Health Organization added Oropouche virus disease to its watch list of emerging arboviral threats [26].

**Figure 1:**
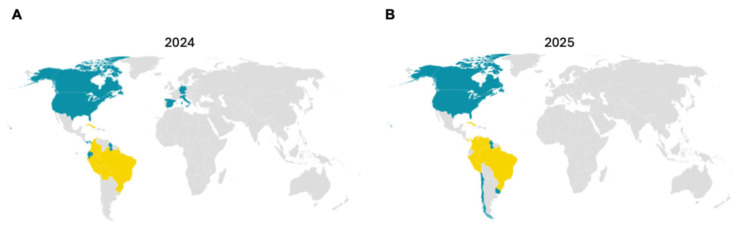
Global reporting status of Oropouche virus (OROV) in 2024 and 2025. (**A**) (2024) and (**B**) (2025) depict countries or regions with reported OROV cases. Yellow indicates autochthonous (locally transmitted) cases. Blue denotes imported cases or those with an unknown transmission route. Gray represents areas with no reported cases. The maps illustrate an expansion in geographic reporting of autochthonous OROV transmission from 2024 to 2025, suggesting an increasing risk of local establishment in previously unaffected or import-only regions. This trend may reflect changes in vector distribution, climate suitability, or human mobility patterns contributing to viral spread. Maps were generated using Datawrapper. Data were compiled from WHO reports, national surveillance systems, and published epidemiological sources.

Oropouche virus (OROV) has exhibited a marked expansion in its geographic range across the Americas during the past two years. Table 2 summarizes the countries affected in 2024 and 2025 [30,31], the corresponding epidemiological categories, and the types of transmission reported. This table outlines the transition from localized endemic foci in South America to widespread regional circulation involving multiple Central American and Caribbean countries. To complement the tabular data, Figure 1A,B visualizes the spatial evolution of OROV distribution between 2024 and 2025.

### 2.2. Transmission Dynamics and Ecological Constraints

**Figure 2:**
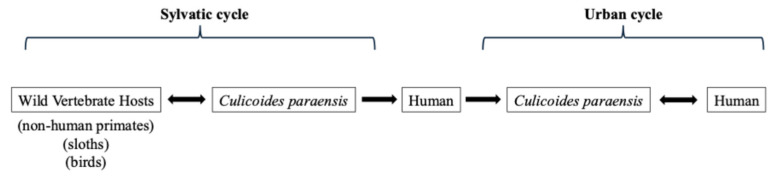
Transmission dynamics of Oropouche virus (OROV) in sylvatic and urban cycles. In the sylvatic cycle, OROV circulates between wild vertebrate hosts (e.g., non-human primates, sloths, and birds) and the biting midge Culicoides paraensis. Humans become infected through spillover following bites from infected midges. In the urban cycle, infected humans serve as amplifying hosts, and transmission occurs between humans via Culicoides paraensis, enabling sustained human–vector–human transmission.

OROV circulates in two ecological cycles. In the sylvatic cycle, the virus is maintained between wild vertebrate hosts, which include sloths, monkeys and birds, and biting midges (*Culicoides paraensis*) (i.e., the principal vector) [1,3,33]. Humans become infected when they encroach on forested habitats or when infected midges adapt to peri-urban areas. The urban cycle is characterized by human-to-human amplification via midge bites, making it the only known Orthobunyavirus with sustained urban transmission [34]. Although *Cx. quinquefasciatus* mosquitoes and other *Culicoides species* have been found to harbour viral RNA, their vector competence remains limited [35,36]. Environmental factors such as flooding, changes in river courses, an increase in temperature, and expanding agriculture create ideal breeding sites for midges and thus increase human risk [37,38]. The transmission dynamics of OROV are illustrated in Figure 2.

Oropouche virus (OROV) is primarily transmitted by the biting midge *Culicoides paraensis* and, to a lesser extent, by mosquitoes such as *Culex quinquefasciatus* [39]. These vectors play a crucial role in defining the virus’s ecological boundaries and its potential for spread beyond the Americas. While *C. paraensis*, which is the principal vector responsible for sustained human transmission, has not been detected in Asia, several related *Culicoides* species capable of transmitting other arboviruses are widely distributed across Southeast and East Asia [39,40]. Surveys have documented diverse *Culicoides* populations in southern China, particularly in Yunnan and Guangxi provinces, as well as neighboring Southeast Asian countries, where warm, humid, and forested environments provide suitable ecological conditions for their proliferation [41,42]. However, no confirmed populations of *C. paraensis* or closely related members of its lineage have been reported in East Asia to date [42], suggesting that natural OROV transmission cycles are currently ecologically improbable in this region.

Despite the absence of competent vectors, the close travel and trade connections between Brazil and China through aviation, academic exchange, and commercial routes could theoretically facilitate the introduction of OROV via infected travelers [43,44]. Travel-associated cases have already been documented in Europe and the United States following exposure in endemic regions such as Brazil and Cuba, highlighting the virus’s capacity for intercontinental movement [44]. Nevertheless, in the absence of established *C. paraensis* populations, imported OROV infections in Asia would likely remain sporadic and self-limiting, without potential for sustained local transmission [40]. According to WHO risk assessments, the current global risk of OROV emergence is considered “moderate” only in areas where competent vectors are present, a condition that does not currently apply to East or Southeast Asia [25].

## 3. Clinical Manifestations and Public Health Impact

### 3.1. Clinical Manifestations and Disease Course of Oropouche Fever

Oropouche virus infection typically causes Oropouche fever, an acute, self-limited febrile illness characterized by high fever, headache, myalgia, arthralgia, and photophobia. Cases occur predominantly during the rainy season, coinciding with increased vector breeding [8]. The incubation period of Oropouche fever is not precisely defined, though field observations during major outbreaks suggest it ranges from 4 to 8 days [8]. Symptoms usually last three to eight days before resolving spontaneously [45]. Nevertheless, approximately 60% of patients experience a second febrile episode one to two weeks after recovery, suggesting a tendency toward symptom recurrence following apparent recovery [46,47]. The disease presents with nonspecific systemic symptoms similar to other arboviral infections such as dengue or Zika, which often leads to diagnostic confusion [46,47,48]. According to a recent systematic review and meta-analysis of 806 laboratory-confirmed Oropouche virus infections across Latin America, the pooled prevalence of clinical symptoms indicated that fever (100%) and headache (95%) were almost universal. Myalgia (72%) and arthralgia (58%) were also frequent, followed by retro-orbital pain (56%), dizziness (45%), and photophobia (31%). Gastrointestinal symptoms such as anorexia (59%), nausea or vomiting (44%), and abdominal pain (28%) were commonly reported, while rash or pruritus occurred in fewer than 15% of cases. Neurological manifestations are uncommon (<2%) and are largely limited to isolated reports of aseptic meningitis and encephalitis, predominantly affecting children and immunocompromised individuals [49,50]. Although mortality is uncommon, the recurrence of symptoms and occasional neurological sequelae contribute to productivity loss and increased healthcare demand in endemic regions [3].

### 3.2. Public Health Implications

Although Oropouche fever is typically self-limiting [6], it poses a substantial public health burden through its combined effects on surveillance, labor productivity, and health system capacity. Its clinical similarity to dengue, chikungunya, and Zika frequently leads to misdiagnosis and underreporting, which obscures the true scale of infection [2]. In many endemic countries, surveillance systems are concentrated in major urban centers, leaving rural and forested areas underrepresented in national statistics [34]. During large outbreaks in Brazil and Peru, health facilities often report a surge of nonspecific febrile illnesses, of which a significant proportion are later confirmed as OROV infections [6,51].

Recurrent febrile episodes further amplify this burden by contributing to absenteeism and productivity loss, particularly in agriculture and informal labor sectors. Studies from the Brazilian Amazon estimate that each epidemic cycle affects thousands of workers, with economic costs comparable to moderate dengue outbreaks [8,52]. In addition, climate and environmental changes such as deforestation, urban expansion, and rising humidity have increased vector density and facilitated the spread of *Culicoides paraensis* into peri-urban areas [6,23,52]. This ecological shift may increase the risk of OROV spread into new regions, including densely populated urban corridors and cross-border zones in northern South America, thereby expanding the population at risk and the associated public health burden.

From a policy perspective, OROV illustrates the challenges faced by low-resource health systems dealing with neglected arboviral diseases [3]. Limited laboratory capacity delays confirmation, and the absence of rapid diagnostic tests hampers timely outbreak response [6,23]. In the absence of specific antiviral therapy or a licensed vaccine, clinical management of Oropouche fever remains supportive. Consequently, public health control efforts depend mainly on vector control and community education measures, such as insecticide spraying, breeding-site elimination, and public awareness campaigns [53]. However, these interventions are often implemented reactively during outbreaks, vary greatly across municipalities, and suffer from limited financial and logistical support, making them difficult to sustain in the long term. The absence of integrated vector management programs and the low public perception of Oropouche fever further reduce the effectiveness of these traditional control strategies [6,23,25]. Given the expanding ecological range of OROV and its overlapping symptomatology with other arboviruses, there is a pressing need for integrated surveillance, standardized diagnostic protocols, and investment in vaccine research to mitigate future outbreaks.

## 4. Vulnerable Populations and True Infection Burden

### 4.1. Definition of Populations Vulnerable to Underrecognized OROV Infection

In the context of Oropouche virus (OROV) infection and vaccine development, vulnerability should be understood not only in terms of clinical severity but also in relation to exposure risk and likelihood of detection. These dimensions together shape the extent to which infections are captured by surveillance systems and, consequently, how the true infection burden is estimated [3,23]. Biological vulnerability includes individuals at higher risk of severe disease or complications due to host factors such as age, immunocompromised status, or pregnancy, which may affect the development and maintenance of protective immune responses and clinical outcomes [3,23,54]. Socioenvironmental determinants such as access to healthcare, geographic isolation, and patterns of occupational or population movement can further shape vulnerability [2,55]. Individuals living in remote rural or forested endemic areas face an increased risk of exposure to Oropouche virus. In these settings, limited access to medical services increases the likelihood that infections remain undiagnosed [56]. Similarly, people living or working in high-vector-density regions, such as peri-urban interfaces or outdoor and agricultural environments, are more frequently exposed and less likely to be captured by routine surveillance [2,8]. Accordingly, vulnerability in the context of OROV extends beyond traditional clinical risk groups to include populations disadvantaged by environmental and socioeconomic conditions.

### 4.2. Vulnerable Populations and Implications for Vaccine Development

From a vaccine development perspective, vulnerable populations require specific considerations during vaccine evaluation and implementation. Table 3 presents a comprehensive summary of clinically vulnerable populations at risk for Oropouche virus (OROV) infection, highlighting representative groups, key biological characteristics, and diagnostic challenges. Newborns and young children under five years old exhibit immature innate and adaptive immunity with low neutralizing antibody levels, leading to diagnostic challenges and uncertainty regarding vertical transmission [57,58]. Elderly individuals (≥65 years) experience immunosenescence, reduced T-cell function, and frequent comorbidities, which contribute to slower viral clearance and higher risks of neurological complications [59,60,61,62]. Pregnant women face hormonal and immune modulation (Th2 bias) that increases risks of fetal death, miscarriage, and congenital anomalies [63,64,65]. Diagnostic testing is complicated and time-consuming, and risk of vertical transmission remains unclear. Immunocompromised patients, including HIV-positive individuals and transplant recipients, show impaired antibody generation and prolonged viremia, with persistent infection and atypical clinical presentations [54,66]. Individuals with chronic metabolic or cardiovascular conditions have systemic inflammation and altered cytokine signaling, increasing their likelihood of severe disease and complications. Lastly, outdoor or agricultural workers in tropical and peri-urban high-vector zones experience high exposure to the *Culicoides paraensis* vector, frequent reinfection risk, and limited healthcare access [39]. Together, these observations highlight that vulnerability to OROV infection arises from both biological susceptibility and patterns of exposure. Table 3 summarizes the major vulnerable populations and their key biological and clinical characteristics.

### 4.3. Gaps in Surveillance and the Underestimated Burden of OROV Infection

The true extent of Oropouche virus (OROV) infections is likely much higher than reported. This is because the symptoms of OROV infection are often very general such as fever and malaise which can easily be mistaken for diseases like dengue or chikungunya [2,71]. During the 2024 arboviral season in Brazil, investigations revealed considerable diagnostic overlap between dengue and OROV infections, with a proportion of suspected dengue cases later confirmed as OROV after laboratory testing [72]. In addition, many infections are mild or asymptomatic and therefore never reach clinical attention. Limited access to laboratory diagnostics in many endemic regions further contributes to under-detection [73]. Together, these factors suggest that official case counts represent only a fraction of the true infection burden.

Evidence from serological studies supports this underestimation. Across the Americas, reported OROV seroprevalence varies widely, ranging from below 5% in urban populations with limited exposure to biting midges to as high as 60% in remote Amazonian communities where Culicoides paraensis vectors are abundant [51]. Occupationally exposed groups, including agricultural and forestry workers, miners, and residents of forest-fringe areas, often show higher seroprevalence than urban populations, highlighting the influence of environmental and behavioral risk factors [48,74]. These findings indicate that OROV transmission is more widespread than suggested by routine case reporting and closely follows ecological gradients of vector density and human exposure.

This discrepancy between reported cases and serological evidence complicates efforts to accurately characterize the epidemiology of OROV. Current surveillance systems remain largely passive and are biased toward acute symptomatic infections, overlooking the substantial proportion of mild or asymptomatic cases that may contribute to viral circulation [6]. In addition, differences in study design and sampling strategies across regions limit direct comparison of seroprevalence estimates [31]. Strengthening surveillance through coordinated serological monitoring and improved diagnostic capacity will therefore be important for clarifying transmission patterns and estimating population-level exposure to OROV.

## 5. Current Landscape of OROV Therapeutics and Vaccines

Although Oropouche virus (OROV) has long been recognized as a neglected tropical arbovirus, therapeutic and vaccine research targeting it remains in its infancy. Several antiviral compounds have demonstrated inhibitory activity in cell culture and animal models, including acridone derivatives, quercetin, and favipiravir, all of which suppress viral replication to varying degrees [6,53]. Most recently, the ribonucleoside analog 4′-fluorouridine (4′-FlU) showed strong in vivo efficacy in murine OROV infection, significantly reducing viral loads and mortality even when administered after symptom onset [75]. These results represent the first demonstration of post-exposure therapeutic potential for OROV, though translation to human use has not yet been achieved. While antiviral development remains important, particularly for post-exposure management, it does not substitute for preventive strategies in populations at sustained risk of infection. At present, all treatment remains supportive, underscoring the need for continued antiviral research, especially for populations with limited access to hospital care.

According to the WHO R&D Blueprint roadmap for Oropouche virus, no OROV-specific vaccine candidates have yet advanced to completed clinical trials up to January 2026 [76]. No OROV vaccine has been licensed, though multiple technological platforms are under investigation, including live-attenuated (BeAn19991 strain) [3,77], viral-vector, protein subunit, mRNA, nanoparticle (NP), and virus-like particle (VLP) vaccines [3,45,53,65,78,79,80]. Animal studies have shown promising immunogenicity and protective effects, and the BeAn19991 candidate has exhibited good tolerability without serious adverse events in preliminary testing. However, no candidate has entered formal human trials. Future vaccine design will need to account for safety considerations in pregnant individuals, immunocompromised hosts, and other vulnerable groups, as well as practical constraints related to deployment in low-resource settings.

## 6. Challenges in OROV Vaccine Development for Vulnerable Populations

Developing an Oropouche virus (OROV) vaccine remains challenging due to several scientific and methodological constraints. These include limited understanding of protective immunity, the lack of representative animal models, gaps in antigen characterization, and persistent diagnostic and surveillance limitations. Together, these factors continue to slow the translation of OROV vaccine concepts into clinical development.

### 6.1. Immunological Considerations

A fundamental challenge in OROV vaccine development is the limited understanding of human immune responses to infection. Existing studies provide only fragmentary insights into innate responses, while adaptive immunity, particularly neutralizing antibody dynamics and T-cell specificity, remains poorly characterized [81,82]. No established correlates of protection (CoP) exist, making it unclear which immunological markers actually confer protection from infection or clinical disease [81]. This gap complicates antigen selection, trial endpoint definition, and immunobridging strategies. For vulnerable populations, the consequences of this uncertainty are magnified. For instance, young children possess immature innate and adaptive immunity, making it unclear how they would respond to a novel vaccine. Similarly, elderly adults, facing immunosenescence, may require higher antigen doses or adjuvanted formulations. Without robust CoP data in these groups, clinical trial design and immunobridging strategies remain highly uncertain.

### 6.2. Lack of Validated and Representative Animal Models

Another major scientific barrier to OROV vaccine development is the absence of reliable animal models that accurately reproduce human disease phenotypes. The WHO and numerous reviews indicate that non-human primates may serve as vertebrate hosts for OROV in nature [83]. However, experimental evidence demonstrating human-like disease phenotypes in these animals remains limited. Most studies only mention the existence of these animals as natural hosts, and there is insufficient experimental evidence to support their use in evaluating protective efficacy. Therefore, available evidence suggests that their value in such research is limited. Moreover, immunodeficient mouse models (such as IFNAR−/− strains) require profound disruption of innate immune signaling to manifest severe disease after OROV infection, whereas wild-type mice remain largely resistant. The disease in IFNAR−/− mice is characterized by high viral replication, hypercytokinemia, liver failure, and rapid lethality, a profile much more severe than in healthy animals or typical human infections [84]. Consequently, the model does not adequately reflect the immune landscape of healthy or vulnerable humans.

The absence of suitable animal models also limits investigation of vaccine responses in specific physiological contexts, including pregnancy, early childhood, and immunocompromised states. Pregnant women and fetuses may be at risk of vertical transmission, yet no model reliably captures maternal–fetal dynamics or placental susceptibility. Likewise, infants and young children, who may exhibit higher viral loads and distinct immune responses, lack pediatric-relevant models that could inform dosing and safety evaluation. Immunocompromised individuals, who often show atypical disease progression [85], are also poorly represented in current experimental systems, which typically rely on artificially induced immunodeficiency rather than clinically realistic immune impairment. As a result, safety and protective efficacy cannot be adequately evaluated in the populations most in need of vaccination, which introduces regulatory uncertainty and slows the translation of promising vaccine candidates into clinical testing.

### 6.3. Antigen Selection, Platform Gaps, and Preclinical Constraints

Compared to dengue, yellow fever, and Zika, Oropouche virus (OROV) vaccine research remains underdeveloped. Only one historical attenuated candidate, the BeAn19991 strain, reached early-phase clinical evaluation, while current candidates, such as vesicular stomatitis virus (VSV) vectored constructs, subunit vaccines, and immunoinformatics-designed peptides, are limited to preliminary animal studies. The lack of high-resolution structural data for OROV surface glycoproteins (Gn/Gc) complicates antigen selection, making it unclear whether vaccines should primarily target Gn/Gc, nucleocapsid (N) protein, or multiple antigens simultaneously [52].

### 6.4. Diagnostic Uncertainty, Surveillance Gaps, and Clinical Trial Barriers

As discussed above, the clinical manifestations of OROV infection overlap extensively with those of dengue, chikungunya, and Zika virus [86], leading to numerous missed diagnoses and underestimation of the actual incidence rate. OROV was not included in routine surveillance in most countries until 2023–2024, resulting in a highly incomplete current disease burden estimate [25,30,31]. These surveillance and diagnostic gaps complicate case confirmation, attack-rate estimation, and clinical trial design, thereby hindering vaccine development.

Limited diagnostic capacity and uneven access to laboratory testing in endemic regions further complicate disease detection and case confirmation. In many rural and peri-urban settings, laboratory infrastructure and trained personnel remain scarce, leading to underdiagnosis and delayed reporting. These limitations hinder accurate estimation of transmission intensity and complicate the evaluation of vaccine efficacy in clinical studies.

## 7. Strategic Framework for Implementation and Equitable Access

Although OROV infection is generally self-limiting and mortality is relatively low, decisions regarding vaccine development should be considered in the context of broader public health priorities. In many endemic settings, strengthening surveillance systems, vector control, and diagnostic capacity may provide substantial benefits for outbreak management. In this context, vaccination strategies may be most relevant for specific high-risk populations or regions with recurrent transmission, and their role would likely depend on context-specific assessments of disease burden and cost-effectiveness.

Considering the scientific and operational constraints outlined above, effective implementation and equitable access to a future Oropouche virus (OROV) vaccine will require coordinated planning and collaborative frameworks tailored to endemic areas. These challenges are particularly evident in remote Amazonian and peri-urban regions where health-system capacity remains limited. Vulnerable groups such as children, elderly adults, pregnant women, immunocompromised individuals, and agricultural workers face elevated exposure risks and barriers to healthcare access. Lessons from yellow fever vaccine campaigns in the Brazilian Amazon, malaria RTS,S pilot programs in sub-Saharan Africa, and HPV vaccination efforts in rural Latin America highlight the importance of decentralized, community-integrated delivery systems [87,88,89,90].

Given the limited commercial attractiveness of OROV vaccines, reliance solely on market-driven forces is unlikely to sustain ongoing research and development. Therefore, establishing a long-term, diversified funding framework is necessary. Drawing from experiences with other neglected tropical disease vaccines, public sector entities, international organizations, and regional financing mechanisms have played pivotal roles in advancing vaccine research, development, and deployment. For instance, the RTS,S malaria vaccine program illustrates how public–private partnerships can support vaccine development and implementation in resource-limited settings [91].

Financing mechanisms such as the PAHO Revolving Fund and WHO prequalification pathways may provide useful models for reducing financial barriers and supporting future vaccine production and scale-up [89]. Similarly, strengthening laboratory diagnostic capacity, molecular surveillance networks, and public health coordination may facilitate both vaccine trials and future immunization programs in endemic settings [76,91]. In addition, post-marketing safety surveillance would likely be important for detecting rare adverse events and evaluating vaccine effectiveness across different regions and population groups [76]. Achieving these goals will require coordination among national health ministries, regional research networks, international organizations such as PAHO, WHO, and CEPI, and private-sector partners.

## 8. Vaccine Platform Suitability for Vulnerable Populations

### 8.1. General Principles

Selecting appropriate vaccine platforms for Oropouche virus (OROV) requires consideration of both biological risk and practical deployment constraints. As discussed in Section 4 and Section 6, OROV disproportionately affects several vulnerable populations, including pregnant women, infants and young children, occupationally exposed adults, elderly individuals, and people with immunocompromising conditions or major chronic comorbidities. These groups differ in baseline risk for OROV disease, susceptibility to severe outcomes, and tolerance of vaccine-related reactogenicity, which together shape platform selection and programmatic use. Given the early stage of OROV vaccine development, platform suitability for these populations must currently be assessed primarily based on general vaccine safety principles and experience with related viral vaccine platforms.

Across these populations, several cross-cutting principles emerge. Non-replicating platforms such as inactivated whole-virus vaccines, recombinant protein or virus-like particle (VLP) vaccines, and potentially mRNA or non-replicating viral vectors are generally preferred because they have a clearer theoretical safety profile and can be tailored in dose and adjuvant content [92]. Live-attenuated OROV vaccines, while potentially highly immunogenic, may pose unacceptable theoretical risks in pregnancy and in severely immunocompromised hosts and should therefore be considered with particular caution [93]. An overarching objective is to identify one or two core platforms that can be adapted formulation-wise to meet the distinct needs of each vulnerable group, thereby simplifying development and deployment.

### 8.2. Vaccine Platform Considerations Across Vulnerable Populations

Although non-replicating vaccine platforms are broadly applicable, biological and immunological differences among vulnerable populations influence their optimal use and immunization strategies.

Pregnant women represent an important target group because OROV infection during pregnancy may lead to maternal illness, placental infection, and potential adverse fetal outcomes [63]. In this context, vaccination strategies should prioritize safety and the induction of maternal neutralizing antibodies capable of efficient transplacental IgG transfer [94]. Inactivated or recombinant protein-based vaccines are therefore particularly attractive theoretical candidates for maternal immunization, as these platforms have well-established safety profiles in other maternal vaccination programs [93].

For infants and young children, vaccine design should account for immune system maturation and the possible influence of maternally derived antibodies. Maternal antibodies may protect infants early in life but can also interfere with vaccine-induced responses [95]. As a result, pediatric vaccine schedules may need to account for the timing of maternal vaccination, the age at first pediatric dose, and the interval between booster doses to optimize immune responses.

In older adults, immunosenescence can reduce both the magnitude and durability of vaccine-induced immunity [96]. Strategies such as higher antigen doses or additional booster doses may therefore be necessary to achieve adequate protection [97,98]. Similar considerations may apply to individuals with major chronic diseases, whose immune responses can be modestly attenuated but generally remain compatible with standard non-replicating vaccine platforms [99].

Immunocompromised individuals require particular caution because impaired immune responses may reduce vaccine effectiveness, while replicating vaccines could pose safety risks [100]. In this population, inactivated or recombinant protein-based vaccines are likely to represent the safest options, although additional doses or tailored schedules may be required to achieve sufficient immune protection.

Taken together, these considerations suggest that a small number of adaptable non-replicating platforms, particularly inactivated and recombinant protein or virus-like particle (VLP) vaccines, could plausibly form the backbone of future OROV vaccination strategies across vulnerable populations.

### 8.3. Operational and Deployment Considerations

Beyond biological and immunological considerations, operational factors related to vaccine delivery may also influence platform suitability. OROV transmission occurs primarily in tropical regions where vaccine delivery capacity may be limited [23,101]. These conditions can complicate both clinical trials and large-scale vaccination programs. Vaccine platforms with simplified logistical requirements may therefore offer practical advantages in endemic settings. Formulations requiring fewer doses, improved thermostability, or reduced cold-chain dependence could facilitate vaccine deployment in remote or resource-limited areas [102]. For example, thermostable or lyophilized vaccine formulations may help mitigate transport and storage challenges in tropical environments.

Integrating OROV vaccination into existing health programs may further enhance feasibility. Potential delivery channels include maternal immunization services and routine childhood vaccination schedules. Aligning OROV vaccination strategies with established immunization platforms could improve coverage while minimizing additional health system burden.

### 8.4. Research Priorities

Despite these conceptual considerations, several important knowledge gaps remain. Key uncertainties include the correlates of protective immunity against OROV, the safety and effectiveness of vaccination during pregnancy, the magnitude of maternal antibody interference in infants, and the optimal antigen dose and booster strategies for elderly or immunocompromised populations [76]. Addressing these questions will require carefully designed clinical studies and post-licensure evaluations across diverse demographic groups.

Ultimately, the development of an effective OROV vaccine portfolio will depend not only on advances in vaccine platform technologies but also on improved epidemiological surveillance, standardized immunological assays, and a clearer understanding of population-specific risks [76]. Strengthening these research areas will be essential for translating candidate vaccines into effective public health interventions.

## 9. Conclusions

Accumulated evidence reviewed here indicates that Oropouche virus (OROV) has transitioned from a geographically confined, sylvatic arbovirus to an emerging pathogen of regional and international concern. However, its true disease burden remains underestimated because infections are frequently misdiagnosed as other arboviral diseases and surveillance and diagnostic capacity remain limited in many endemic regions. The evidence reviewed here suggests that vulnerability to OROV infection is shaped by the interaction of epidemiological exposure, biological susceptibility, and unequal access to healthcare, which may increase risk among populations such as young children, older adults, pregnant individuals, and immunocompromised persons.

Although OROV vaccine development remains in an early stage, multiple vaccine platforms have demonstrated preclinical promise. Advancing these candidates will require addressing several scientific and practical challenges, including a limited understanding of protective immunity and logistical constraints in endemic settings. Importantly, vaccine development strategies should consider the specific needs of vulnerable populations when selecting appropriate platforms and deployment approaches. At the same time, effective prevention of OROV will require not only advances in vaccine development but also strengthened surveillance and improved diagnostic capacity.

## Figures and Tables

**Table 1:** Historical Phases of Oropouche Virus (OROV) Emergence and Spread (1955–2025).

Phase	Time Period	Key Events/Features	Quantitative Data (Cases)	Geographic Range
Phase I—Discovery and Sylvatic Cycle Recognition	1955–1960	1955—First human case in Vega de Oropouche, Trinidad (strain TRVL 9760) [10] 1960—Isolation from *Coquillettidia venezuelensis* mosquitoes, Nariva Swamp, Trinidad [10] 1960—Detection in sloths and *Aedes serratus* mosquitoes, Pará, Brazil [8]	—	Trinidad and Tobago; Northern Brazil (Pará)
Phase II—Endemic establishment in the Amazon Basin	1961–1979	1961—Belém outbreak linked to Belém–Brasília highway construction [11] 1961–1979—≥4 epidemics documented in Pará [11]	~11,000 (1961) [11] >60,000 cumulative (1961–1979) [11,13]	Pará, Brazil
Phase III—Regional Expansion in Northern South America	1980–1999	1980s—≥30 epidemics across Acre, Amapá, Maranhão, Rondônia, and Tocantins; expansion beyond Pará; regional endemic circulation in northern Brazil [11] 1992—First detection in Peru (Iquitos) [14,15]	>100,000 (1980–1981) [13,16].	Northern and Western Brazil, Panama, Peru
Phase IV—Continental Expansion and Reservoir Identification	2000–2010	2000—Genotype III identified in wild marmoset (*Callithrix penicillata*), SE Brazil [17]; Identification of animal reservoirs and host adaptation 2000–2007—New regions of infection reported in Ecuador and Bolivia [14,18]; Expansion beyond Amazon Basin; cross-border spread	—	Brazil (widespread), Ecuador, Bolivia
Phase V—Urbanization and Silent Circulation	2011–2019	2011–2016—3% positivity in arbovirus-negative sera (Amazonas) [19] 2015–2016—Outbreaks in San Martín, Cajamarca, Cusco (Peru) [20] 2016—First coastal Brazil case (Bahia); OROV RNA detected in serum, saliva, and urine [21] 2019–2022—Two independent introductions confirmed in Colombia [22]	3% positivity (9/306 samples, 2011–2016 Amazonas) [23]	Brazil (Amazonas, Bahia); Peru; Colombia
Phase VI—Regional Re-emergence and International Spread	2020–2025	2020—French Guiana outbreak; dengue-like clinical presentation [24] 2023—Major epidemic in northern Brazil [25] 2024—Regional spread to Peru, Bolivia, Ecuador, Colombia, Cuba, Panama [25]; Imported cases reported in the U.S. and Europe [25,26]; Recognition as emerging arbovirus of regional concern	23/28 PCR positive (82.1%)—French Guiana, 2020 [24] >16,107 confirmed cases—Americas, 2024 [27] 45,456 confirmed cases—Americas EW1-30 [26].	Latin America; imported cases in North America and Europe

**Table 2:** Epidemiological status of Oropouche virus in the Americas, 2024–2025.

Country/Region	Status (2024)	Cases (2024)	Deaths (2024)	Transmission (2024)	Status (2025)	Cases (2025)	Deaths (2025)	Transmission (2025)
Brazil	Endemic—Expanding	13,785	4	Widespread local (Amazon + non-Amazon states)	Endemic—Expanding	11,888	5	Sustained local across 23 states
Peru	Endemic	1263	0	Local transmission (Amazon departments)	Endemic	330	0	Local transmission (Amazon departments)
Bolivia	Emerging	356	0	Local transmission (La Paz, Beni, Pando)	—	—	—	—
Colombia	Emerging	74	0	Local transmission (Amazonas focus)	Emerging	26	0	Local transmission
Cuba	Newly Affected	626	0	Autochthonous urban transmission	Endemic (continued circulation)	28	0	Local urban transmission
Panama	—	—	—	—	Emerging	501	1	Local transmission (Darién Province)
Venezuela	—	—	—	—	Emerging	5	0	Local transmission
Guyana	—	—	—	—	Emerging	1	0	Local transmission
Ecuador	Historical	—	—	—	—	—	—	—
United States	Imported	1	0	Imported (from Cuba)	Imported	1	0	Imported (from Panama/Brazil)
Canada	Imported	2	0	Imported (from Cuba)	Imported	1	0	Imported (from Colombia)
Chile	—	—	—	—	Imported	2	0	Imported (from Brazil)
Uruguay	—	—	—	—	Imported	3	0	Imported (from Brazil)
Europe	Imported	30	0	Imported (from Cuba/Brazil)	Imported	≈30	0	Imported (from Brazil/Cuba)

Notes: Epidemiological categories are defined as follows. “Endemic” indicates sustained local transmission in historically affected regions; “Emerging” indicates recently reported local transmission; and “Imported” refers to infections acquired outside the reporting country. “—” indicates that no confirmed data or no updated information was available in the cited reports. Confirmed cases refer to laboratory-confirmed Oropouche virus (OROV) infections detected by RT-PCR or validated serological assays. Case and death counts represent reported totals for 2024; 2025 data reflect PAHO/WHO epidemiological updates available as of 13 August 2025. Data source: Data compiled from PAHO and WHO epidemiological situation reports and updates on Oropouche virus in the Americas [31,32]. Data for 2025 are based on epidemiological updates available up to 13 August 2025 [32]; sources accessed October 2025.

**Table 3:** Summary of Clinically Vulnerable Populations for OROV Vaccine Development.

Category	Representative Population	Key Biological Features	Clinical or Diagnostic Challenges
Newborns and Young Children	Infants and children under 5 years old	Immature innate and adaptive immunity; low neutralizing antibody levels [57,58]	Non-specific febrile symptoms; high likelihood of misdiagnosis with dengue or chikungunya; risk of vertical transmission uncertain for newborns [63,67,68]
Elderly Individuals	Adults ≥ 65 years	Immunosenescence; reduced T-cell function; frequent comorbidities [59,60,61,62]	Slower viral clearance; higher neurological complication risk [62,69]
Pregnant Women	Women during pregnancy	Hormonal and immune modulation (Th2 bias); fetal death, miscarriage and congenital anomalies [63,64,65]	Diagnostic testing difficulty [68]; risk of vertical transmission uncertain [63]
Immunocompromised Patients	HIV-positive individuals, transplant recipients, or those on immunosuppressive therapy	Impaired antibody generation; prolonged viremia [54,66]	Persistent infection and higher viral load; atypical presentation [54,66]
Comorbidity-Related Groups	Individuals with chronic metabolic or cardiovascular conditions	Systemic inflammation; altered cytokine signaling [70]	Higher probability of severe disease and complications [70]
Outdoor or Agricultural Workers	Field, forestry, and construction workers; residents in high-vector zones	High exposure to *Culicoides paraensis* in tropical and peri-urban areas [39]	Frequent reinfection risk; limited healthcare access [39]

## Data Availability

No new data were created in this review article.
